# Parainfluenza Virus Type 1 Induces Epithelial IL-8 Production via p38-MAPK Signalling

**DOI:** 10.1155/2014/515984

**Published:** 2014-06-11

**Authors:** Miguel Ángel Galván Morales, Carlos Cabello Gutiérrez, Fidencio Mejía Nepomuceno, Leticia Valle Peralta, Elba Valencia Maqueda, María Eugenia Manjarrez Zavala

**Affiliations:** Department of Virology and Mycology Research, National Institute of Respiratory Diseases “Ismael Cosio Villegas”, Calzada de Tlalpan 4502, Colonia Sección XVI, Delegación Tlalpan, 14080 México, DF, Mexico

## Abstract

Human parainfluenza virus type 1 (HPIV-1) is the most common cause of croup in infants. The aim of this study was to describe molecular mechanisms associated with IL-8 production during HPIV-1 infection and the role of viral replication in MAPK synthesis and activation. An *in vitro* model of HPIV-1 infection in the HEp-2 and A549 cell lines was used; a kinetic-based ELISA for IL-8 detection was also used, phosphorylation of the mitogen-activated protein kinases (MAPKs) was identified by Western blot analysis, and specific inhibitors for each kinase were used to identify which MAPK was involved. Inactivated viruses were used to assess whether viral replication is required for IL-8 production. Results revealed a gradual increase in IL-8 production at different selected times, when phosphorylation of MAPK was detected. The secretion of IL-8 in the two cell lines infected with the HPIV-1 is related to the phosphorylation of the MAPK as well as viral replication. Inhibition of p38 suppressed the secretion of IL-8 in the HEp-2 cells. No kinase activation was observed when viruses were inactivated.

## 1. Introduction

Human parainfluenza virus type 1 (HPIV-1), which is a member of the family Paramyxoviridae, is an enveloped virus with a single-stranded RNA genome [1, 2]. HPIV-1 infects the upper and lower respiratory tract and causes acute respiratory infections (ARIs), ranging from mild infections, such as the common cold and laryngitis, to severe infections, such as croup, pneumonia, and bronchiolitis. HPIV-1 is responsible for almost half of all hospitalizations due to ARIs both in patients younger than 5 years old and in the elderly; additionally, HPIV-1 is the most common cause of infectious laryngotracheitis (croup) in children [3–6]. The therapy used to treat symptoms of inflammation is based on glucocorticoid and ephedrine, also humidifying the airway; however, this is not always effective [7–9]. The pathogenic mechanisms activated by HPIV-1 during infection are largely unknown. Local response mechanisms have been described in which innate and adaptive defence systems participate. There is no evidence indicating that mitogenic signal activation is required in the early stages of infection [10, 11]. IL-8 is a mediator responsible for the recruitment of neutrophils that participate in the local inflammatory infiltrate, contributing to airway closure [12, 13]. It has been reported that epithelial cells, alveolar macrophages, and neutrophils secrete IL-8 [14–17]. Other authors have reported that infection with respiratory syncytial virus (RSV), varicella-zoster virus, and smallpox virus activates IL-8 secretion without viral replication [18, 19]. These observations indicate that the interaction between the virus and its receptor is sufficient to promote the signalling pathways that activate the IL-8 gene [20]; however, replication is necessary in other viruses, such as vaccinia virus and rhinovirus [21–23]. It has been shown that viruses have different effects on the regulation of IL-8 expression and secretion. The most prominent examples include the filoviruses Marburg and Ebola and arenaviruses, such as Lassa and Junin. Other viruses such as RSV, adenovirus, vaccine virus, and herpes virus secrete IL-8 immediately [24, 25].

The primary signalling pathways that elicit a response by chemokines are the MAPKs and transcription factor NF kappa B pathways. MAPKs are a family of proteins that activate kinases through a cascade of intracellular phosphorylation events and signal transduction from the cell surface to the nucleus. They are composed of three well-characterized subfamilies: extracellular signal-regulated kinases (ERK1/2), c-Jun N-terminal kinases (JNKs), and p38 mitogen-activated proteinkinases (p38). Each subfamily includes a kinase that sequentially acts on three proteins: MEKK, MEK, and MAPK. Each protein is activated through phosphorylation. The MAPK family substrates in the cytoplasm and nucleus include additional kinases, transcription factors, phospholipases, and cytoskeletal proteins. ERK1/2 is associated with anabolic processes, such as cell division, growth, and differentiation, while JNK and p38 are associated with cellular responses to stress conditions, death, and inflammation [26–28].

The molecular mechanisms in which larynx epithelial cells play a role and their active involvement in the inflammatory infiltration response to infection by HPIV-1 through production of small chemoattractants that recruit neutrophils have not been investigated sufficiently to generate a strategy that counteracts pathogenesis and to determine whether viral replication is necessary. In this study, an* in vitro* model of HPIV-1 infection of HEp-2 and A549 cells was used to simulate the upper and lower airways. The aim of this study was to determine whether viral replication induces the IL-8 secretion and MAPK kinase signalization.

## 2. Materials and Methods

### 2.1. Cell Lines

The HEp-2 human laryngeal epithelioma type 2 cells (ATCC CCL-23, USA, which reported a contamination with HeLa cells) and A549 human alveolar type II-like epithelial cells (ATCC, CCL-185, USA) were acquired from American Type Culture Collection (Manassas, VA, USA). Cell lines were propagated in 25 cm^3^ flasks (Nunc Thermo Scientific Inc., Turnberry, USA) and Petri dishes with Eagle's Minimal Essential Medium (MEM), supplemented with 10% foetal bovine serum and antibiotics (Penicillin 100 U/mL, Streptomycin 100 *μ*g/mL, Amphotericin-B 1 *μ*g/mL) from Invitrogen Life Technologies (Gaithersburg, MD). Cells were maintained at 37°C in 5% CO_2_ and were stored and grown in accordance with the manufacturer's instructions.

### 2.2. HPIV-1 Propagation

Virus strain was obtained from American Type Culture Collection, Manassas, VA, USA. (ATCC number VR-94, strain: C35 HPIV-1). Confluent monolayers of HEp-2 and A549 cells in 25 cm^3^ flasks were inoculated with HPIV-1 and incubated at 37°C in 5% CO_2_ until a cytopathic effect was observed 48 hours after inoculation (formation of syncytia). The virus was titrated by plaque assay using methylcellulose. One aliquot of the virus was transferred to 1.8 mL cryotubes after titration and stored at −70°C, and another aliquot was used to infect cell monolayers at a 0.01 multiplicity of infection (MOI). Uninfected HEp-2 and A549 cell monolayers were used as controls.

### 2.3. HPIV-1 Inactivation

A portion of HPIV-1 (5 mL of virus at a titre of 6 × 10^6^ PFU) in serum-free medium (RPMI) was placed in a sealed box and irradiated at a distance of 10 cm for 10 minutes with optimal radiation levels (1,200 units × 100 *μ*J/cm^2^) using an FB-UVXL 1000 UV cross-linker Fisher Biotech (Thermo Fisher Scientific Inc., KY, USA) as previously described [29]. Inactivation was verified by plaque assay on monolayers of HEp-2 and A549 cells. Haemagglutination assays were performed in 96-well plates to verify the haemagglutinin activity of HPIV-1 using serial dilutions of O-positive human blood (1 : 2, 1 : 4, etc.) [30]. Results are expressed in hemagglutinating units (HAU) [31, 32].

### 2.4. Quantitation of IL-8

HEp-2 and A549 cells were infected with live and inactive HPIV-1 at an MOI of 0.01, supernatant was collected at different times (30, 45, 60, 90, and 120 minutes and 12 and 24 hours), and uninfected HEp-2 and A549 cells were used as controls. IL-8 concentrations were quantitated by ELISA according to the procedure described by Kittigul et al. (1998) [33] using anti-human CXCL8/IL-8 monoclonal antibodies from R&D Systems, Inc. (Minneapolis, MN, USA). Optical density at 450 nm was measured on an ELISA reader spectrophotometer from Thermo Labsystems (Santa Rosa, CA, USA, and Helsinki, Finland).

### 2.5. Reverse Transcription-Polymerase Chain Reaction

The expression of mRNA for IL-8 was determined by reverse transcription-polymerase chain reaction (RT-PCR). Total RNA was isolated by using SV Total RNA Isolation System (Promega, Madison, USA) following the manufacturer's protocol. A 0.5 *μ*g portion of total RNA was reverse-transcribed for subsequent PCR amplification for each pair of primers in a volume of 20 *μ*L, including 200 U M-MLV RT reverse transcriptase (Invitrogen, CA, USA), 50 U of RNase inhibitor (Sigma), 0.2 *μ*g of random primer, 10 mM of dNTP Mix (Invitrogen, CA, USA), and 5x First-Strand Buffer (250 mM Tris-HCl, pH 8.3; 375 mM KCl; 15 mM MgCl_2_) provided by Life Technologies. The reaction was incubated at 42°C for 50 min. A 20 *μ*L portion of the RT products was then brought to a volume of 20 *μ*L containing 10 mM of each dNTP, 1 U of Taq polymerase (Promega, Madison, USA), 10 pmol of both the upstream and downstream PCR primers, and 1 x PCR buffer (proprietary formulation supplied at pH 8.5 containing blue dye and yellow dye) provided by Promega. The primers for IL-8 are as follows: forward, 5′-ATG ACT TCC AAG CTG GCC GTG GCT-3′ and reverse, 5′-TCT CAG CCC TCT TCA AAA ACT TCT-3′ with a product of 289 bps [34]. For glyceraldehyde-3-phosphate dehydrogenase (GAPDH); the forward primer was 5′-CCAGCCGAGCCACATCGCTC-3; and the reverse primer was 5′-ATGAGCCCCAGCCTTCTCCAT-3′, giving a 390 bps PCR product. The GAPDH gene was amplified in the same reaction to serve as the reference gene. Amplification was carried out in a Biometra personal thermal cycler after an initial denaturation at 94°C for 3 min. This was followed by 35 cycles of PCR using the following temperature and time profile: denaturation at 94°C for 40 s, primer annealing at 58°C for 40 s, primer extension at 72°C for 1 min, and a final extension of 72°C for 6 min. The PCR products were visualised by electrophoresis on a 2% agarose gel and visualized by UV transillumination.

### 2.6. Detection of MAPK Phosphorylation (ERK1/2, JNK, and/or p38)

HEp-2 and A549 cells were infected with live or inactive HPIV-1 at an MOI of 0.01 with the same kinetics as that described above in the previous assays. Cells were harvested, and total protein was extracted and quantified by the Bradford method. Cells were boiled at 100°C for 10 minutes in a buffer solution supplemented with *β*-mercaptoethanol. The obtained proteins were separated on 15% SDS-PAGE gels and transferred to a 0.45 *μ*m nitrocellulose membrane from Bio-Rad Laboratories (Hercules, CA) for 3.5 hours, and Western blot assays were performed. Transfer efficiency was determined by staining with 0.1% Ponceau red in 5% glacial acetic acid from Sigma-Aldrich (St. Louis, MO), and measurements were performed using Laemmli's SDS-PAGE system [35]. Membranes were washed, blocked, and probed overnight with MAPK phospho-ERK1/2 (sc-81492), MAPK ERK1/2 (sc-292838), phospho-p38 MAPK (sc-7973), p38 MAPK (sc-535), phospho-JNK (sc-135642), and JNK (sc-571) antibodies (1 : 3 000 dilution) purchased from Santa Cruz Biotechnology (Santa Cruz, CA). The bound antibody was coated with a peroxidase-conjugated goat anti-mouse IgG secondary antibody (Sigma-Aldrich) (1 : 3 000) for 2 hours. Membranes were washed and incubated with a luminal chemiluminescent substrate from Santa Cruz Biotechnology, before exposure to X-ray film. The band intensity was quantified using the Quantity One (Bio-Rad) Image Analysis software.

### 2.7. IL-8 Quantitation in the Presence of Kinase Inhibitors

For the best reproduction of larynx conditions, assays were conducted using HEp-2 cells that were pretreated with the kinase inhibitors PD980559 for ERK1/2, SB203580* for p38 MAPK, and SP600125* for JNK from Calbiochem Millipore* (San Diego, CA). The concentrations used were from 5 to 20 *μ*M (1.3 to 5.2 *μ*L) in DMSO; the same DMSO concentration was added to control cells and was incubated at 37°C. One mL of HPIV-1 at an MOI of 0.01 was added, and infected cells were incubated under the same conditions. Supernatants were collected at the selected times, and IL-8 concentrations were determined. Supernatants from HEp-2 cells without inhibitors collected at the same infection time were used as controls. When the MAPK inhibitor was used, cells were pretreated with the inhibitor 1 hour before infection. Because MAPK inhibitors were diluted in DMSO, equal concentrations of DMSO were added to the untreated infected cells as control. After treatment with the inhibitor, IL-8 concentrations were measured and supernatant was collected at the different times tested.

### 2.8. Quantitation of the Bands

The analysis done in the Western blot bands was performed in the Quantity One software from Bio-Rad. Individual quantification of the bands was made from the intensity of light generated in them, due to the fluorescence emitted in the radiographic plate.

### 2.9. Statistical Analyses

Data were tested for normality (Shapiro-Wilk) and since data did not show normal distribution, Kruskal-Wallis test was used to compare the different treatments. The assays were performed in triplicate, and the results were statistically analysed using Statistica Software 5.0 (Stat Soft, Tulsa, Oklahoma). A *P* value <0.05 was considered to indicate a statistically significant difference.

## 3. Results

### 3.1. Evaluation of HPIV-1 Inactivation

The inactivated viruses were created to demonstrate whether viral replication was involved in the activation of the secretion of IL-8 or viral structure alone was sufficient to generate the response to this chemokine. The virus was inactive with UV radiation; plaque assays were performed to verify that the virus was found inactive and will not be replicated.

**Figure 1:**
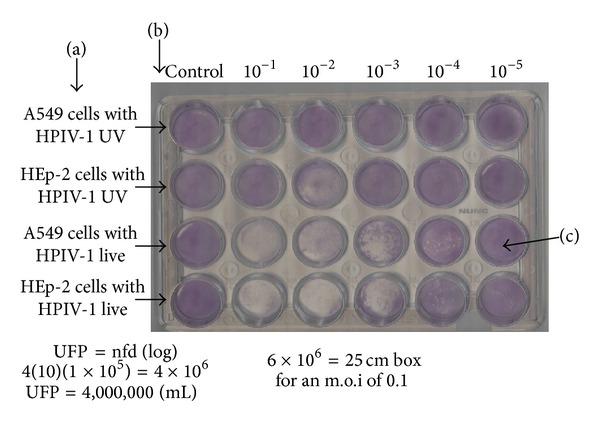
Lytic plaque assay. The number of viral particles was determined by dilution. In a 24-well plate with HEp-2 and A549 cell cultures, each lytic plaque that develops is initially related to a single infectious virion particle. This method was performed to determine the viability of both viruses, live and inactive. (a) refers to the cell type and the characteristic of the virus (inactive and live); (b) corresponds to dilutions ranging from 10^−1^ to 10^−5^; and (c) refers to plaque-forming units. This assay was performed in triplicate.

Exponential virus dilutions are presented in Figure 1 to determine the number of infectious particles. (a) The first two rows correspond to the viral activity with the inactive virus, where there was no replication due to the absence of PFU. In the remaining rows of live virus activity in both cell types was evaluated. Panel (b) corresponds to viral dilutions and panel (c) refers to the number of infective particles in a dilution type cellular.

**Figure 2:**
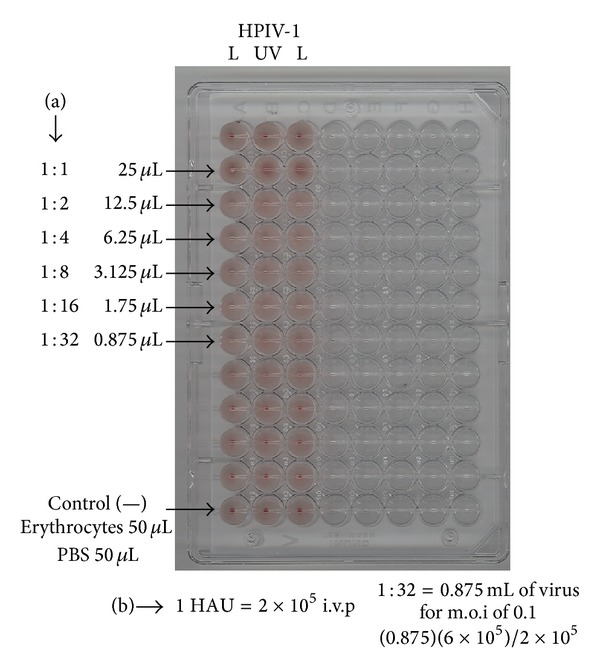
Haemagglutinating capacity of both live and inactive virus was determined in a 96-well plate. (a) Serial dilutions of the virus: 1 : 2, 1 : 32; (b) hemagglutinating units (HAU). L corresponds to the live virus lane, and UV corresponds to the inactive virus. This assay was performed in triplicate.

Because the inactive virus did not replicate, haemagglutination tests were made showing that inactive virus particles had the same ability to agglutinate erythrocytes and the active virus (L). In both lytic and haemagglutination assays exponential dilutions were prepared to determine the virus viability (Figure 2).

These tests are often used to demonstrate the viral replication, for the presence of hemagglutinin glycoprotein or viable structures.

### 3.2. IL-8 Secretion in Cell Lines Infected with Live and Inactive Virus

IL-8 secretion was quantified during HPIV-1 infection suggesting that infection of cell lines with live HPIV-1 induced a gradual increase in IL-8 secretion. The highest IL-8 secretion in HEp-2 and A549 was observed at 12 hours after infection, whereas the cells with inactive virus showed a marginal response at 24 hours (2.05 and 2.0). The higher secretion of IL-8 was found using infected HEp-2 cells followed by infected A549 cells (Table 1), compared with the cells with inactive virus where a minimal response was achieved. In the case of HEp-2 cells and A549 cells treated with inactive virus, no IL-8 secretion was observed neither in the uninfected cells used as controls.

### 3.3. Reverse Transcription-Polymerase Chain Reaction

**Figure 3:**
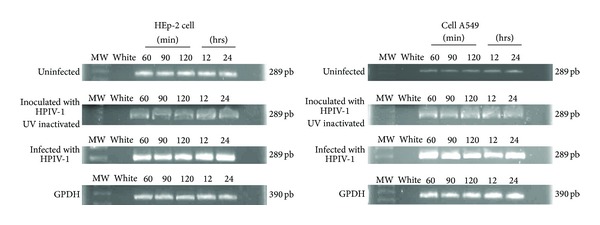
IL8 expression by RT-PCR analysis in HEp2 and A549 cells infected, uninfected, and the HPVI-1 inactive. The enhanced expression of IL8 was given in infected cells HPVI-1. GAPDH expression was not different by infection.

The mRNA expression of IL-8 in A549 and HEp-2 cells was determined by RT-PCR analysis. In the analysis, uninfected cells, cells infected with HPIV-infected cells, and HPIV-1 inactive were used. The increased expression of IL-8 originated from infected cells, followed by uninfected cells and the inactivated virus, and the expression in the latter two was almost equal. Infection was performed at different times and behavior for each was similar in both cell lines (Figure 3). GAPDH was constitutively expressed in both cell lines and expression did not vary by infection.

### 3.4. HPIV-1 Infection Stimulates MAPK Phosphorylation

**Figure 4:**
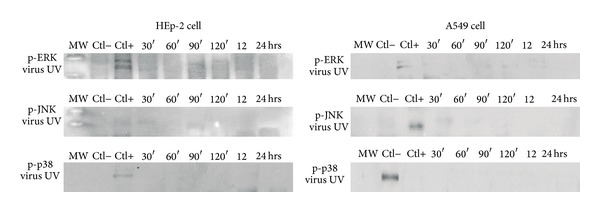
Kinetics of expression of MAPKs in HEp-2 and A549 cells after inoculation with HPIV-1 virus inactive. ERK1/2, JNK, and p38 phosphorylation showed no time points analyzed during 30 min to 24 hrs. Ctl 2nd lane (−) refers to the proteins of uninfected cells. The Ctl (+) corresponds to the proteins from cells infected with live virus (90 min). Antibodies used are described in Materials and Methods. Western blot assays were performed in triplicate.

To establish whether the inactive virus stimulated the phosphorylation of MAPKs (ERK, JNK, and p38) Western blot tests were made (Figure 4). The two different cell lines HEp-2 cell line for the upper portion of the tract and the A549 for the bottom line of the same tract were used to simulate the respiratory tract.

**Figure 5:**
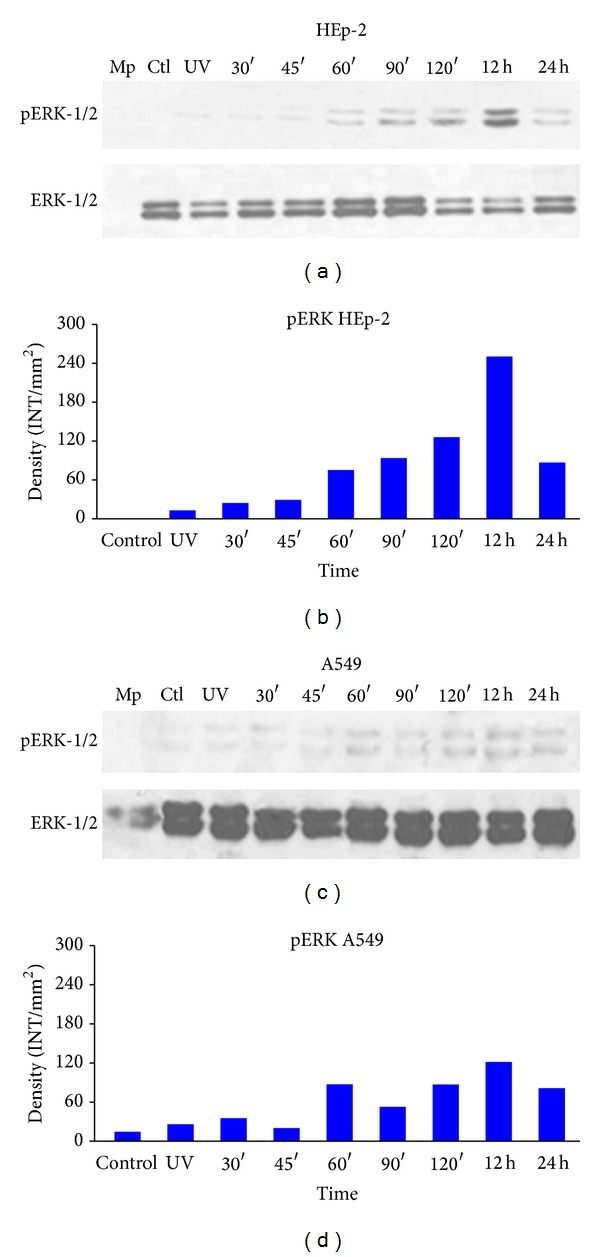
ERK phosphorylation.Detection of phosphorylation of ERK1/2 by Western blot in protein extracts of HEp-2 and A549 cells infected with HPIV-1 live at different time points: (a) phospho-ERK was detected with p-ERK1/2 (sc-81492) antibody as a loading control and total ERK1/2 (sc-292838) in HEp-2 cells; (b) densitometric analysis of ERK1/2 protein concentrations; (c) phospho-ERK and total ERK1/2 protein concentrations in A549 cells; (d) densitometric analysis of protein concentrations. Mp: molecular weight; CTL: control and UV (inactive virus) that corresponds to 12 hours in path 3. Data are the means for triplicate measurements from the three separate experiments.

Western blot assays were performed to determine the phosphorylation status of the MAPKs ERK1/2, JNK, and p38. In HEp-2 cells, infection with live HPIV-1 promoted early ERK1/2 phosphorylation, beginning at 60 minutes of interaction (Figures 5(a) and 5(b); band intensities were quantified through densitometric analysis). Phosphorylation increased at 90 and 120 minutes. However, ERK phosphorylation was higher at 12 hours than at any other selected time and decreased at 24 hours. In the case of inactivated virus, the 12-hour band was only in lane 3 because no evidence of phosphorylation was observed in the kinetics selected times. The same criteria were applied to the Western blot figures; only a band corresponding to a 12-hour interaction was observed with no increase in phosphorylation before and after this time point. Controls consisted of uninfected cells, which showed no significant changes.

In A549 cells infected with live HPIV-1, the detection of ERK1/2 phosphorylation began at 60 minutes of interaction; a small phosphorylation signal was observed at 90 minutes of interaction (Figures 5(c) and 5(d)), increasing slightly at 120 minutes and remaining constant until the 24-hour time point. Phosphorylation was slightly detected for the inactivated virus and control samples. In controls (uninfected cells), inactive virus samples, and samples that were incubated for 30 and 45 minutes phosphorylation was only slightly detected. With inactive viruses, the result was similar to the previous Western blot (Figures 5(c), lane 4, and 5(d), lane 3).

**Figure 6:**
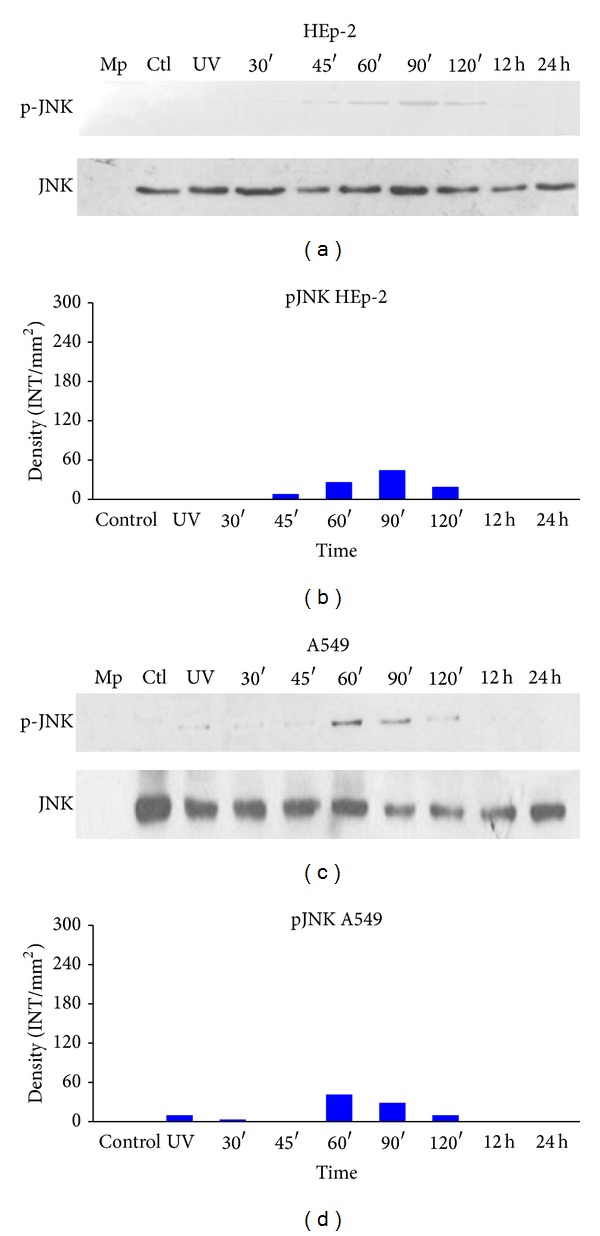
JNK phosphorylation. Detection of JNK phosphorylation in HEp-2 and A549 cells infected with HPIV-1. Cells were infected with live and inactive HPIV-1 at different time points, and JNK phosphorylation was detected. Whole cell lysates were prepared for Western blotting (as described in Materials and Methods) to detect the phosphorylated (active) forms of the kinases: (a) phospho-JNK was detected with monoclonal antibody sc-135642 and the total JNK sc-571 in HEp-2 cells; (b) densitometric analysis of JNK protein concentrations; (c) phospho-JNK and total protein concentrations in A549 cells; (d) densitometric analysis of the protein concentrations. Mp: molecular weight; CTL: control and UV (inactive virus) that corresponds to 12 hours in path 3. Data are the means for triplicate measurements from the three separate experiments.

JNK phosphorylation in HEp-2 cells also began at 60 minutes and gradually increased until the 90-minute time point but decreased at 120 minutes. By 12 and 24 hours, phosphorylation ceased (Figures 6(a) and 6(b)). As in HEp-2 cells, JNK phosphorylation in A549 cells began at 60 minutes, increased at 120 minutes, and decreased at 12 and 24 hours, with no further increase (Figures 6(c) and 6(d), lanes 9 and 10).

**Figure 7:**
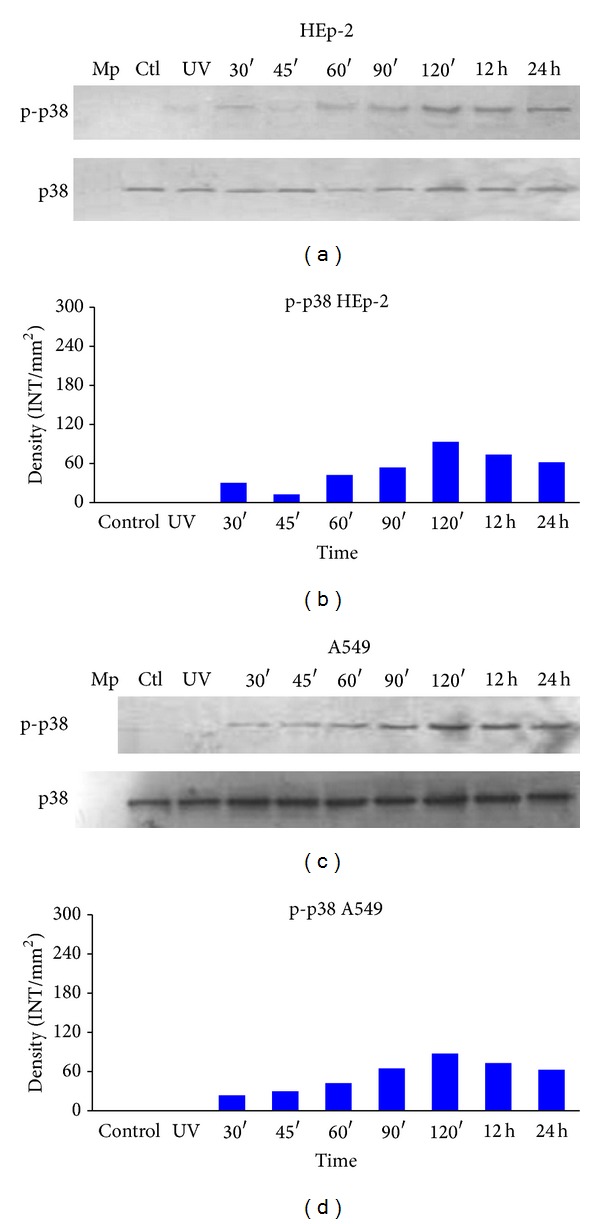
p38 phosphorylation. Detection of p38 phosphorylation in HEp-2 and A549 cells infected with HPIV-1. Cells were infected with live and inactivated HPIV-1 at different time points, and p38 phosphorylation was detected. Whole cell lysates were prepared for Western blotting (as described in Materials and Methods) to detect the phosphorylated (active) forms of the kinases. (a) Total protein concentrations in HEp-2 cells. Phospho-p38 was detected with monoclonal antibody sc-7973 and the total p38 sc-535 in HEp-2 cells; (b) densitometric analysis of p38 protein concentrations; (c) phospho-p38 and total protein concentrations in A549 cells; (d) densitometric analysis of the protein concentrations. Mp: molecular weight; CTL: control and UV (inactive virus) that corresponds to 12 hours in path 3. Data are the means for triplicate measurements from the three separate experiments.

Likewise, p38 phosphorylation in the HEp-2 cell line began at 30 minutes (Figures 7(a) and 7(b)), increased again at 120 minutes, and subsequently remained stable until the 24-hour time point. In A549 cells, p38 phosphorylation levels were similar, beginning at 30 minutes (Figures 7(c) and 7(d)) but with a gradual increase from the 90-minute time point and achieving a maximum at 12 and 24 hours.

### 3.5. IL-8 Secretion Decreased with HPIV-1 Infection through p38 Inhibition

The kinase inhibition assay was performed on the cell line HEp-2, derived from human laryngeal epithelioma, given that is a suitable target for the* in vitro* virus assay. Our results suggest that p38, ERK1/2, and JNK are activated following HPIV-1 infection. Therefore, to determine whether these kinases are required for IL-8 gene expression, ELISAs were performed using the supernatant from HEp-2 cells pretreated with ERK1/2, JNK, and p38 kinase inhibitors and infected with HPIV-1 at the following time points: 45, 60, and 90 minutes and 12 and 24 hours (Figure 9). Results demonstrate that the p38 inhibitor repressed IL-8 production upon viral infection, whereas ERK1/2 and JNK MAPK inhibitors did not inhibit IL-8 production (Figures 9(a) and 9(b)), suggesting that p38 MAPK signalling is involved in IL-8 production.  Figure 9(c) depicts the average of three experiments with HEp-2 cells infected with HPIV-1 and inhibitors. Controls without inhibitors for each kinase were included for all time points.

Treating the cells with JNK and ERK1/2 inhibitors did not impair IL-8 production, but treating cells with an inhibitor of p38 MAPK significantly impaired IL-8 secretion in most of the incubation times (before 24 h). ERK1/2 and JNK inhibitors were not different from the control group (Table 2).

**Figure 8:**
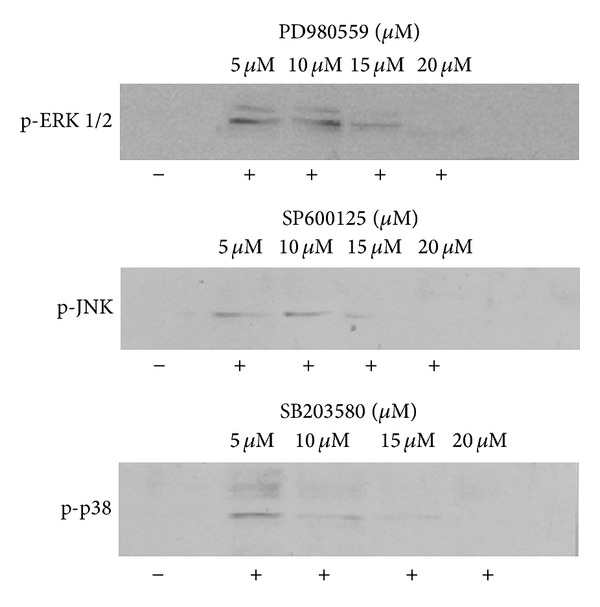
Inhibitors of ERK1/2 PD980559, JNK SP600125, and p38 SB203580 inhibited the expression of each of the MAPKs in the HEp-2 cell line treated cell 1 hour before infection and maintained for 120 minutes after the test. The experiments were performed by adding inhibitor at concentrations from 5 *μ*M to 20 *μ*M (1.3 to 5.2 *μ*L, resp.). Protein levels were detected by Western blot. Immunoblotting was done with monoclonal antibody specific for protein phosphorylated technique that was described in “Materials and Methods”. The extracted proteins (−) represent HEp-2 cells without infecting and the proteins (+) refer to cells infected with the HPIV-1. The tests were performed in triplicate.

**Figure 9:**
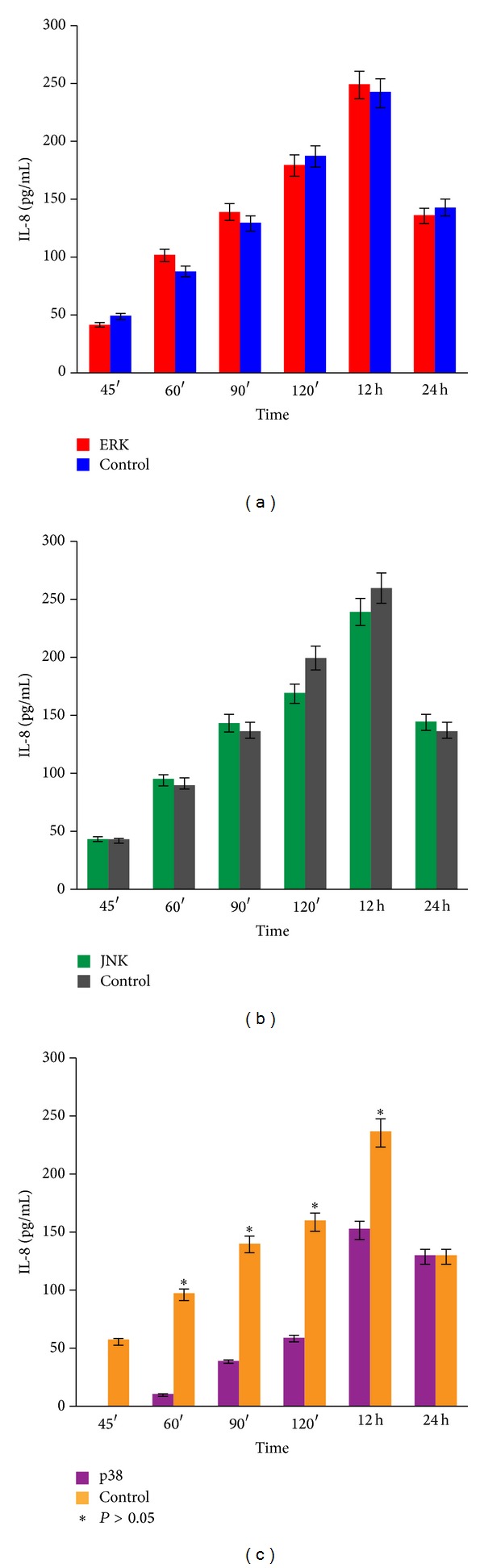
Kinetic inhibition of MAPKs. ELISA quantitation of IL-8 concentrations in cells pretreated with ERK1/2 PD980559, JNK SP600125, and SB203580 inhibitors. Control is HEp-2 cells infected with live HPIV-1. (a) ERK1/2 inhibitor assay is displayed in red cells treated with the inhibitor. (b) JNK inhibitor assay, green, occurs in cells treated with the inhibitor. (c) p38 inhibitor assay, purple, occurs in cells treated with inhibitor. Data are the means ± SD for triplicate measurements from the three separate experiments.

Total inhibitory dose MAPKs (ERK1/2, JNK, and p38) were evaluated, showing that the optimal concentration to suppress phosphorylation was 20 *μ*M (Figure 8).

## 4. Discussion

In the present study, an* in vitro *model with two cell lines that were infected with HPIV-1 at different time points was used to determine whether an increase in IL-8 secretion occurred. Our assay showed that IL-8 secretion gradually increased during the time points assessed.

These results are consistent with reports that other respiratory viruses, including* influenza A virus* (H3N2) and RSV, induce IL-8 secretion from bronchial epithelial cells [36, 37].

It is known that IL-8 is responsible for the recruitment of neutrophils and macrophages and that it performs other functions, such as adhesion and degranulation. Likewise, the presence of IL-8 in inflammatory infiltrates in lung tissues has been reported. Studies performed with different viruses have revealed that epithelial cells infected with specific viruses release IL-8 without requiring viral replication, as what occurs in RSV infections [18, 19]. Contact with the cellular receptor is sufficient to trigger IL-8 secretion, whereas other viruses only promote IL-8 synthesis during replication [21, 23]. In this study, assays using both live and inactivated HPIV-1 were performed to determine whether replication-independent IL-8 release occurs with HPIV-1, a virus from the same family as RSV. The results indicate that contact between the virus and target cell is insufficient, even when haemagglutination activity is intact in the inactivated virus, and viral replication is required to stimulate IL-8 secretion.

Previous reports have shown that IL-8 secretion is involved in MAPK pathway activation [12, 16]. These kinases respond to different ligands and stimuli and are involved in signalling pathways and cellular processes, such as proliferation, differentiation, inflammation, immune response, and apoptosis.

HPIV-1 does not use the JNK pathway for IL-8 activation, whereas p38 and ERK are phosphorylated when IL-8 is present in the supernatant after infection.

The results of this study showed that HPVI-1 does not use the JNK pathway for activation of IL-8, while the MAPKs p38 and ERK are phosphorylated by the production of IL-8 after the infection, similar results have been described after RSV infection (virus belonging to the Paramyxoviridae family) in human lung endothelial cells where ERK phosphorylation enabled IL-8se creation [37].

Therefore, to define which kinases are required for IL-8 secretion, the same assays were performed using specific inhibitors for each kinase. The results showed that, even in the presence of specific inhibitors of ERK and JNK, IL-8 was still secreted, but there was no secretion in the presence of p38 inhibitor. In this case, a complete suppression of IL-8 secretion was observed, which suggests that p38 MAPK is the pathway through which HPIV-1 induces IL-8 production.

It is known that the inhibitor SB203580 is a potent, cell-permeable, selective, and reversible inhibitor of p38*α* and *β* isoforms. Inhibition is competitive with ATP and requires substrates to accommodate the fluorophenyl ring structure of the pyridinyl imidazole in their ATP-binding pocket. Structures of other p38 isoforms, JNKs, and ERK1/2 do not allow inhibitor binding at equivalent positions [38, 39].

Many members of the Paramyxovirus family have been shown to be potent inducers of chemokines of the CXC family such as interleukin-8 (IL-8), including respiratory syncytial virus (RSV), Sendai virus, human parainfluenza virus type 2 (HPIV-2), HPIV-3, and Newcastle disease virus [40].

Paramyxoviruses have two glycoproteins that are important during infection. The receptor-binding glycoprotein is HN in parainfluenza virus and mumps virus, H in measles virus, and G in pneumoviruses (RSV and metapneumovirus). The second glycoprotein is the fusion protein (F), which catalyses membrane fusion (viral and cellular). It has been suggested that HN glycoproteins form a complex that is initiated by the binding HN, activating the fusion protein that acts immediately in such a way that the virus can enter the cell. However, it must be noted that glycoprotein HN acts as both a haemagglutinin (HA) and a neuraminidase (NA), binding to sialic acid molecules through its HA activity, mediating the enzymatic cleavage of sialic acid and promoting fusion through its neuraminidase activity. This dual activity suggests that coexpressed HN and F form a complex. Glycoprotein H of morbillivirus only has haemagglutinin activity, and glycoprotein G has neither of these two activities [41–44]. Therefore, these glycoproteins exhibit significant differences. Such differences are likely related to signalling pathways that are activated when viruses interact with the cell. Although HPIV-1 and RSV belong to the same family, the two viruses have different glycoproteins, which may explain why RSV uses ERK kinase and HPIV-1 uses p38 kinase. Following virus binding to its cellular receptor, the extracellular stimulus is transmitted to the nucleus to activate the expression of various genes involved in a given biological activity. The virus activates these pathways according to the coevolution of the parasite-host complex, and it is likely that the virus also can take advantage of the activation of the pathway to provide a better environment and thus freely replicate. Viruses, such as herpesviruses [12], hepatitis B virus [11], HIV [45], influenza virus [36], Coxsackie virus, and vaccinia virus, activate the MAPK pathway, particularly ERK, in the early stages of infection. In this study, we found that MAPKs are activated during the early stages of infection (60 minutes). It is possible that the activation of the p38 kinase pathway following HPIV-1 infection plays a similarly essential role during other stages of infection. Importantly, the stimulus by HPIV-1 increased and was maintained up to 24 hours; therefore, it was detectable at the first stages of infection, even when cytopathic effect was clearly visible in at least 90% of the culture. Several authors have asserted that kinase activation could be a requirement for signalling regulation associated with different biological functions during infection with other viruses [22]. For example, the first events during HIV-1 infection depend on the MAPK/ERK pathway, which improves viral infectivity [45].

The mechanism by which HPIV-1 stimulates p38 activation needs to be further investigated. However, a product of any of the virus genes may be involved in the process, considering that no kinase activation was observed when viruses were UV-irradiated. Additionally, it is possible that MAPKs also influence virus replication, promoting the synthesis of some proteins necessary for the virus.

HPIV-1 infection causes inflammation of the larynx and trachea or croup in children. Children with croup are regularly admitted to the emergency department with their lives at risk due to airway closure. Currently, the treatment for patients with moderate to severe croup is oral dexamethasone at 0.6 mg/kg (maximum 10–12 mg) doses, which acts on these and additional cell types involved in the immune response [46]. However, data show that p38 MAPK inhibitors may reverse glucocorticoid insensitivity and have beneficial effects on glucocorticoid concentrations in patients with asthma as well as airway inflammation, in addition to its role as a treatment substitute [47].

A better understanding of HPIV-1 pathogenesis will guide the design of strategies and therapies allowing the inhibition of excessive cell recruitment and IL-8 synthesis to reduce the uncontrolled inflammatory process. For now, the exact mechanism in inflammatory and immune processes must be defined, and further research is required to understand whether these pathways are significant for HPIV-1 pathogenesis.

## 5. Conclusions

An* in vitro* model with two cell lines infected with HPIV-1 at different time points was used to determine whether an increase in IL-8 production occurred. The results showed that IL-8 production gradually increased during the time points assessed and that viral replication is required for IL-8 secretion. It was found that MAPKs are activated during the early stages of infection. No kinase activation was observed when viruses were inactivated, suggesting that a product of any of the virus genes may be involved in the process. When a p38 inhibitor was used, IL-8 concentrations decreased, suggesting that HPIV-1 induces IL-8 secretion through p38. Additionally, it is possible that MAPKs also affect virus replication, promoting the synthesis of some proteins necessary for the virus. However further studies are needed to understand whether these pathways are significant for HPIV-1 pathogenesis, in order to develop strategies and therapies allowing the inhibition of excessive cell recruitment and IL-8 synthesis to reduce the uncontrolled inflammatory process.

## Figures and Tables

**Table 1:** IL-8 secretion (pg/mL) in HEp-2 and A549 cells infected with live and inactive virus.

Time	HEp-2 live virus	A549 live virus	HEp-2 inactive virus	A549 inactive virus	SD	SEM	Chi square	*P*	CV
Uninfected cells	0.004^a^	0.001^a^	0.002^a^	0.027^a ^	0.018	0.005	3.71	0.29	207.49
30′	39.01^b^	47.04^a^	0.02^c^	0.06^c^	22.65	6.54	10.38	0.01	105.16
45′	55.03^b^	62.11^a^	0.03^c^	0.03^c^	30.69	8.86	9.35	0.02	104.74
60′	104.15^a^	92.72^b^	0.01^c^	0.04^c^	51.57	14.89	10.38	0.01	104.74
90′	132.38^a^	135.07^a^	1.33^b^	1.00^b^	69.24	19.99	9.46	0.02	102.65
120′	210.08^a^	201.72^b^	0.02^c^	0.01^c^	107.56	31.05	9.35	0.02	104.47
12 hours	265.72^a^	239.84^b^	0.01^c^	0.01^c^	132.36	38.21	9.43	0.02	104.71
24 hours	145.06^a^	129.30^b^	2.05^c^	2.00^c^	70.83	20.45	9.46	0.02	101.74

^a,b,c^Means with no common superscript in a row differ (*P* < 0.05). SD: standard deviation; SEM: standard error of the mean; *P*: probability value; CV: coefficient of variation.

**Table 2:** IL-8 production (pg/mL) in infected HEp-2 cells treated with ERK1/2, JNK, and p38 kinase inhibitors.

Time	ERK1/2	JNK	P38	Uninfected cells	SD	SEM	Chi square	*P*	CV
45′	41.88^a^	43.71^a^	0.06^b^	50.04^a^	20.94	6.04	8.74	0.03	61.71
60′	102.16^a^	95.27^a^	11.10^c^	92.48^b^	75.26	11.25	9.46	0.02	51.78
90′	139.25^a^	143.91^a^	39.73^b^	135.85^a^	45.51	13.14	8.43	0.03	39.68
120′	179.71^a^	169.71^a^	59.48^b^	181.99^a^	54.30	15.68	7.61	0.05	36.75
12 hours	243.08^a^	240.05^a^	152.78^b^	246.07^a^	41.33	11.93	6.58	0.08	18.74
24 hours	136.39^ab^	144.50^a^	129.51^b^	137.07^ab^	6.50	1.88	7.46	0.05	4.74

^a,b,c^Means with no common superscript in a row differ (*P* < 0.05). SD: standard deviation; SEM: standard error of the mean; *P*: probability value; CV: coefficient of variation.
